# Interdepartmental Spread of Innovations: A Multicentre Study of the Enhanced Recovery After Surgery Programme

**DOI:** 10.1007/s00268-018-4495-z

**Published:** 2018-01-31

**Authors:** Jeanny J. A. de Groot, José M. C. Maessen, Cornelis H. C. Dejong, Bjorn Winkens, Roy F. P. M. Kruitwagen, Brigitte F. M. Slangen, Trudy van der Weijden, on behalf of all the members of the study group, R. L. M. Bekkers, E. A. Boss, E. B. L. van Dorst, W. J. van Driel, G. Fons, K. N. Gaarenstroom, C. G. Gerestein, M. van Haaften, A. M. L. D. van Haaften-de Jong, H. H. de Haan, D. van Hamont, R. H. M. Hermans, W. Hofhuis, L. N. Hofman, J. E. Martens, H. Mertens, B. M. Pijlman, J. M. A. Pijnenborg, N. Reesink-Peters, E. M. Roes, J. H. Schagen van Leeuwen, M. P. L. M. Snijders, P. M. L. H. Vencken

**Affiliations:** 1 Department of Family Medicine, CAPHRI, School for Public Health and Primary Care, Maastricht University, P.O. Box 616, 6200 MD Maastricht, The Netherlands; 2 Department of Obstetrics and Gynaecology, Maastricht University Medical Centre, P.O. Box 5800, 6202 AZ Maastricht, The Netherlands; 3 Department of Quality and Safety, Maastricht University Medical Centre, P.O. Box 5800, 6202 AZ Maastricht, The Netherlands; 4 Department of Surgery, Maastricht University Medical Centre, P.O. Box 5800, 6202 AZ Maastricht, The Netherlands; 5 NUTRIM – School for Nutrition and Translational Research in Metabolism, Maastricht University Medical Centre, P.O. Box 5800, 6202 AZ Maastricht, The Netherlands; 6 GROW – School for Oncology and Developmental Biology, Maastricht University Medical Centre, P.O. Box 5800, 6202 AZ Maastricht, The Netherlands; 7 Department of Methodology and Statistics, CAPHRI, School for Public Health and Primary Care, Maastricht University, P.O. Box 616, 6200 MD Maastricht, The Netherlands

## Abstract

**Background:**

Spread of evidence-based innovations beyond pioneering settings is essential to improve quality of care. This study aimed to evaluate the influence of a national project to implement ‘Enhanced Recovery After Surgery’ (ERAS) among colorectal teams on the spread of this innovation to gynaecological procedures.

**Methods:**

A retrospective observational multicentre study was performed of a consecutive sample of patients who underwent major elective gynaecological surgery in 2012–2013. Ten Dutch hospitals (294 patients) had participated in a colorectal breakthrough project implementing ERAS on a nationwide basis and were assigned to the intervention group. Thirteen hospitals (390 patients) that had not participated in this project acted as controls. Outcome measures were time to functional recovery and total length of postoperative hospital stay. Multilevel models adjusted for clustering and baseline demographics were used for analysis. The uptake of ten selected perioperative care elements was evaluated for each hospital.

**Results:**

The estimated mean difference (95% confidence interval) between the intervention and control hospitals was −0.3 (−0.9 to 0.3) days in the time to recovery and 0.2 (−0.8 to 1.3) days in the total length of hospital stay. The mean (± standard deviation) absolute rate of implemented perioperative care elements per hospital was 28.9 ± 14.9% in the control, versus 29.3 ± 11.1% in the intervention group (*p* = 0.934).

**Conclusion:**

Initial implementation effects seem to be restricted to the participating teams and do not automatically spread to other surgical teams in the same hospital.

## Introduction

Innovations in healthcare are essential to ensure progress and to achieve the highest quality of care [1]. Despite the potential impact on outcomes, change of practice is generally slow and challenging [2]. Quality improvement (QI) programmes are widely used to achieve change by applying a systematic approach. A growing body of evidence on their effectiveness has become available in recent years [3]. QI programmes, such as the collaborative breakthrough method [4, 5], provide support to spread innovations between stakeholders during the improvement process [6]. Spread of health innovations beyond this pioneering setting is necessary to continue improving quality of care. Studies have recognised that spread is also important to sustain innovation effects after an implementation project has ended [7]. Nevertheless, improvements often remain adopted by a restricted group of innovators [8].

Greenhalgh et al. [9] described a continuum between unplanned (diffusion) and planned (dissemination) mechanisms to achieve spread. Following this broad concept, we defined spread as the process through which effective innovations are adopted from one setting to another. Spread of clinical knowledge between departments can be hampered by intra-organisational barriers, such as group membership [10, 11]. A multiple case-study demonstrated that hospitals are able to spread both processes and content to other medical-surgical units using deliberate and active approaches to change (dissemination) [12]. Limited knowledge exists about the full continuum of local spread mechanisms when dissemination is not promoted and supported at a higher, national level.

We hypothesised that the experiences and tools gained after running a QI project among colorectal surgical teams would stimulate spread of a universal perioperative innovation to another closely related intra-organisational department, such as the gynaecological department. To examine this influence, we compared perioperative care and outcomes of gynaecological procedures in hospitals in which the colorectal surgical teams had taken part in a QI project [13], with the procedures performed by gynaecologists in hospitals in which colorectal surgical teams had not participated in the QI project.

## Materials and methods

### Design

A 1-year breakthrough project was used to implement ‘Enhanced Recovery After Surgery’ (ERAS) across 33 colorectal surgery departments in the Netherlands between 2006 and 2009 [13]. A significant change in practice was achieved, resulting in reduced length of hospital stay [13]. The spread from colorectal to gynaecological surgery was selected for analysis in the current retrospective multicentre study. The gynaecology and colorectal surgical teams were closely related and specialists cooperated regularly during surgical procedures, but departments operated independently. This observational study was exempt from ethics review (Medical Ethical Committee of the Maastricht University, METC13-5-031 and METC14-5-083).

### Inclusion criteria

Dutch hospitals authorised to carry out major gynaecological cancer surgery were invited to participate. In the Netherlands, hospitals are required to meet a minimum number of 20 major ovarian cancer surgical procedures per year for authorisation. Both university and non-university teaching hospitals have the capacity to fulfil this criterion. The hospitals that had participated in the colorectal breakthrough project were included in the intervention group. Authorised hospitals that had not participated in an international or national project implementing ERAS for colorectal surgery acted as controls. One hospital participated in an international collaboration to develop and implement ERAS [14] and took the lead as an expert centre in the national implementation of ERAS in colorectal surgery [13]. As such, this hospital did not fulfil the criteria for the intervention or the control group and was excluded. The spread of ERAS towards gynaecology within this pioneering centre and the effects after active implementation have been described elsewhere [15]. The hospitals that regularly refer patients to an extended care facility for inpatient rehabilitation after surgery were excluded to prevent biased outcome measures. Hospitals with a combined ward for gynaecological and colorectal surgery could participate, but were registered as such.

A consecutive sample of patients who underwent elective open surgery were audited. Eligible patients were aged at least 18 years and had a suspected or proven diagnosis of ovarian, uterine, or cervical cancer. Surgical procedures included open exploratory, staging, and cytoreductive procedures in 2012 and 2013.

### Description of the innovation

The uptake of the multifaceted ERAS guideline was evaluated. Based on the best available evidence, ERAS consists of several perioperative recommendations to reduce time of recovery after surgery [16]. Although originally developed for colorectal surgery, the programme is not exclusive to this specialty [17]. To date, more surgical specialties, such as gastric [18] and pancreatic surgery [19], urology [20], and gynaecology [21, 22], have adopted ERAS and have tailored guidelines to specific procedures or to local policies [23]. Individual elements of ERAS, such as the avoidance of drains, have been shown to be effective in gynaecological surgery for several years [24–28].

### Outcome measures

Primary outcomes were the time to functional recovery after surgery (FR) and the total length of postoperative hospital stay (TLOS) in days. Recovery was reached when patients tolerated oral food and oral analgesia and regained their mobility. TLOS included the number of nights a patient stayed in the hospital after surgery plus, if appropriate, the length of readmission. The secondary outcome included the degree of local implementation of single ERAS elements; for each hospital, this was dichotomised using a target of 70% adherence as a minimum degree of implementation [29, 30]. A combined score per element was calculated. Mortality and readmission rates were registered.

### Data collection

Retrospective review of medical records was performed up to 30 days postoperatively. In considering the Dutch volume norm, a maximum of 30 patients per hospital were audited. Hospital types were categorised as university and non-university teaching hospitals. There is one specialised cancer centre, which was categorised as a university hospital. The type of gynaecological cancer, histological subtype, and type of incision were specified. To obtain accurate information, data of ten ERAS elements were registered (Table 1). These elements covered the three perioperative phases of care, and their combination represents an ERAS management.

**Table 1:** ERAS elements included in retrospective analysis

Preoperative phase	Omission of mechanical bowel preparation
	Use of carbohydrate load
Operative phase	Use of local analgesia
	Avoidance of drains
Postoperative phase	Early ambulation on the day of surgery (sitting on edge of bed)
	Scheduled postoperative administration of laxatives
	Oral feeding on POD 1
	Early ambulation on POD 1 (sitting on chair three times a day)
	Discontinuation of intravenous fluids on POD 2
	Discontinuation of urinary catheter on POD 2

*ERAS* Enhanced Recovery After Surgery, *POD* postoperative day

### Data analysis

A descriptive analysis of baseline characteristics was performed. For categorical variables, differences were analysed using the *χ*^2^ test or Fisher’s exact test. The independent-samples *t* test was used for numerical variables. To account for clustering of patients within hospitals, linear multilevel methods were used to evaluate differences in time to FR and TLOS. The models incorporated hospital identification as a random effect, whereas study group, hospital type, presence of a combined ward, age (< 60 versus ≥ 60), American Society of Anaesthesiologists classification, type of cancer, histological subtype, and type of incision were included as fixed effects. Missing outcome values were not imputed, since likelihood-based methods were used. The estimated marginal means (EMM) and mean differences (MD), corresponding 95% confidence intervals (CI), and intra-class correlation coefficients (ICC) were presented. Unadjusted differences in the median time to FR and TLOS were analysed at hospital level using the Mann–Whitney U test because of the relatively small number of hospitals (13 versus 10). A reduction in time to FR and TLOS of more than 1 day was assumed to be clinically relevant. A value of *p* ≤ 0.005 was considered statistically significant. Analyses were performed with IBM SPSS Statistics for Windows, version 21.0 (Armonk, NY, USA).

## Results

**Fig. 1:**
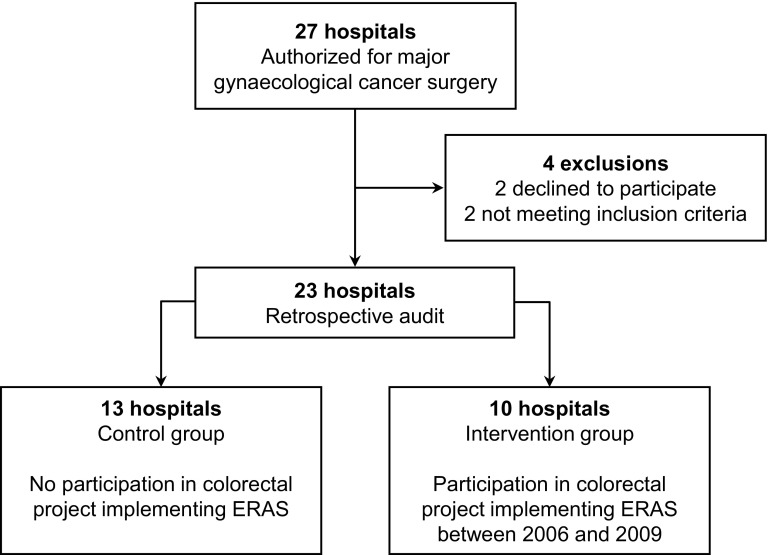
Study flow chart

A total of 684 medical records from 23 hospitals were audited (Fig. 1). Ten hospitals had followed the breakthrough strategy for colorectal surgery and were allocated to the intervention arm. The other hospitals were regarded as controls (*n* = 13). There were no statistically significant between-group differences in demographic data regarding the hospitals (Table 2). Some differences in the indication for surgery were noted between patient groups.

**Table 2:** Baseline demographics of hospitals and patients according to observational group

	Control	Intervention	*p* value^b^
Hospital demographics			
Number of hospitals	13	10	
Number of beds per hospital^a^	642.3 ± 304.5	812.1 ± 189.8	0.138^c^
Type of hospital			0.660^d^
University medical centre	4 (30.8)	2 (20.0)	
Non-university teaching hospital	9 (69.2)	8 (80.0)	
Hospital ward			
Combined with colorectal surgery	1 (7.7)	2 (20.0)	0.560^d^
Patient demographics			
Number of patients	390	294	
Age, years^a^	61.4 ± 12.0	62.6 ± 12.4	0.212^c^
Age, years			0.016
< 60	164 (42.1)	97 (33.0)	
≥ 60	226 (57.9)	197 (67.0)	
ASA classification			0.092
Class I/II	352 (90.3)	253 (86.1)	
Class III/IV	38 (9.7)	41 (13.9)	
Gynaecological cancer type			0.045
Ovarian	289 (74.1)	231 (78.6)	
Uterine	72 (18.5)	54 (18.4)	
Cervical	29 (7.4)	9 (3.1)	
Histological subtype			0.981
Benign	46 (11.8)	36 (12.2)	
Borderline/hyperplasia	30 (7.7)	22 (7.5)	
Malignant	314 (80.5)	236 (80.3)	
Type of incision			0.842
Midline	377 (96.7)	285 (96.9)	
Transverse	13 (3.3)	9 (3.1)	

Values in parentheses are percentages unless indicated otherwise

*ASA* American society of anaesthesiologists

^a^Values are mean ± standard deviation. ^b^*χ*^2^ test, except ^c^independent samples *t* test and ^d^Fisher’s exact test

**Fig. 2:**
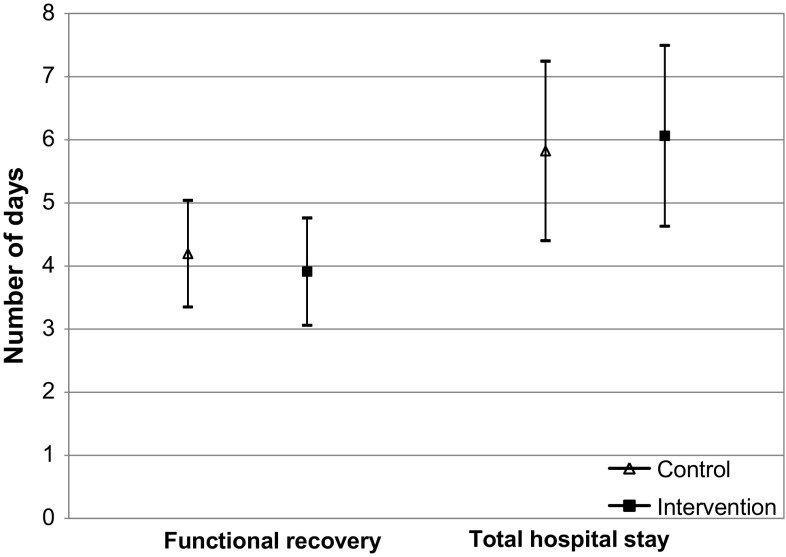
Model estimated marginal means in days

FR was registered for 97.2% of the patients in the control and for 98.6% in the intervention group. Data on the time to FR were missing in five patients in the control and in one patient in the intervention group. In addition, two patients in the control group were transferred to another hospital before recovery was reached. These patients were lost to follow up. Mortality rate was comparable between the control (*n* = 4, 1.0%) and intervention (*n* = 3, 1.0%) groups (*p* > 0.999). The hospitals’ median time to FR was 4.0 days (between-hospital range 3.0–6.0) in the control group, and 4.0 days (between-hospital range 3.0–5.0) in the intervention group (*p* = 0.218). Following adjustment for baseline variables, multilevel modelling showed an MD of −0.3 days (95% CI −0.9 to 0.3) in FR between patients treated in intervention hospitals compared to treatment in a control hospital. The EMM was 4.2 days (95% CI 3.4–5.0) in the control group compared to 3.9 days (95% CI 3.1–4.8) in the intervention group (*p* = 0.319) (Fig. 2). The ICC for FR was 0.03.

The median TLOS was 6.0 days in the control hospitals (between-hospital range 4.0–7.0) and 5.5 days for hospitals in the intervention group (between-hospital range 3.5–10.5) (*p* = 0.777). The observed effect remained statistically nonsignificant after adjusting for covariates (MD 0.2 days; 95% CI −0.8 to 1.3). The EMM was 5.8 days (95% CI 4.4–7.2) in the control group compared to 6.1 days (95% CI 4.6–7.5) in the intervention group (Fig. 2). The ICC was 0.06. In total, 12 patients (3.1%) from the control and 14 patients (4.8%) in the intervention group had to be readmitted (*p* = 0.254).

**Fig. 3:**
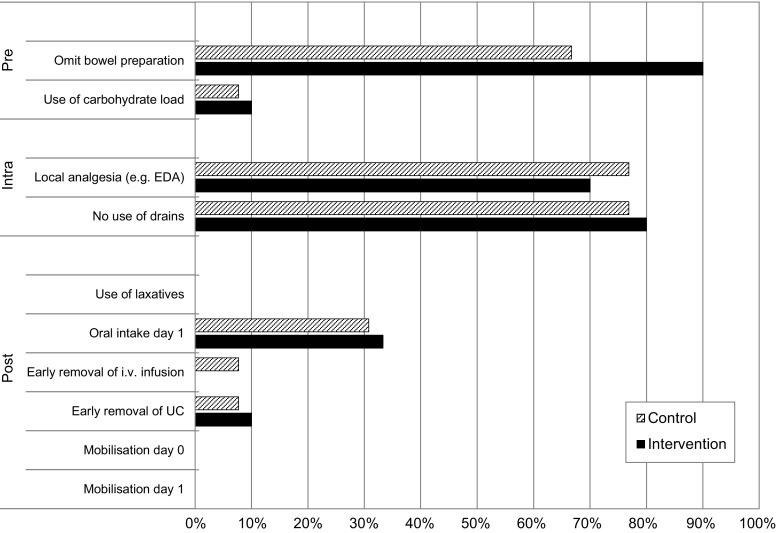
Percentage of implemented (> 70%) enhanced recovery elements among hospitals

The degree of implementation within hospitals was based on the adherence to ten selected ERAS elements. Omission of mechanical bowel preparation, the use of local analgesia, and the avoidance of abdominal drains were the most common elements adhered to in both study groups (Fig. 3). Adoption of postoperative ERAS elements was low. No statistically significant differences were found between groups. In the control group, three hospitals (23%) implemented at least half the elements in daily practice compared to one hospital (10%) in the intervention group (*p* = 0.604). None of these were hospitals with a combined ward for colorectal and gynaecological surgery patients. The mean absolute rate of implemented elements per hospital was 28.9% (SD 14.9%) in the control and 29.3% (SD 11.1%) in the intervention group (*p* = 0.934).

## Discussion

Despite the acquired knowledge and positive experiences gained from the implementation of ERAS in colorectal surgery, this study showed that almost no spread took place in the closely related department of gynaecology in the 5 years following implementation. Multilevel regression analysis, taking clustering of hospitals and baseline demographics into account, demonstrated no differences in outcomes between gynaecological procedures performed in hospitals that had participated in the colorectal QI project, and the hospitals that did not. Even the hospitals with a mixed ward for colorectal and gynaecological surgery did not adopt the majority of ERAS elements.

None of the single ERAS elements was regularly adopted by all the hospitals, despite the clear evidence already available for many years [21, 22]. This finding confirms the outcomes of a single centre study in an early-adopter hospital [15] and emphasises the challenge to spread and actively implement ERAS in actual practice [31]. Greenhalgh and colleagues [9] have described several characteristics of innovations that are required to facilitate spread. Although the multifaceted and behaviour changing aspects of ERAS may render implementation in actual practice difficult, the innovation is particularly suitable to spread within organisations. ERAS principles are universal, and effectiveness has been demonstrated [32–34]. Despite the complex multifaceted structure, implementation can be achieved by using small, manageable steps. Benefits are almost directly visible for both the innovators and patients. Furthermore, the need for a multidisciplinary approach should facilitate intra-organisational spread.

This study included hospitals that participated in the original breakthrough strategy [13]. The breakthrough strategy is designed to spread innovations across multiple organisations over a short period of time [4]. Participants learned to apply practical skills to improve care, and all participating hospitals reduced length of hospital stay and/or had high adherence rates [13]. Therefore, the expertise acquired should give them the capacity to guide local spread. In this study, we tried to analyse the full continuum of local spread without promotion and guidance at a higher, national level. None of the hospitals achieved a complete spread of ERAS, and just one hospital in the intervention group implemented at least half of the ERAS elements. Unfortunately, we lack consistent information about the sustainability of ERAS in colorectal surgery in those hospitals.

Labgaa et al. [35] recently noted that structured implementation of ERAS in one speciality (colorectal surgery) induced a transition of elements towards another speciality (liver surgery) within the same department in patients treated by the same team. Evidence is limited about the effect of conducting a systematic QI programme on the spread of innovations between departments. It has been shown frequently that changing behaviour within healthcare organisations is challenging and complex [2, 3, 36]. Although results need to be interpreted with caution, we believe this study emphasises those findings and shows that barriers are even higher than generally expected. Awareness about the barriers might be the first step in breaking through apparent boundaries and creating a culture of change. During recent years, the shift from a traditional function-oriented towards a process-oriented structure of hospitals has gained attention [37]. The functional grouping of departments in the Netherlands seems to hamper the multidisciplinary efforts to optimise patient care. Breaking through this group membership facilitates knowledge sharing between different groups of healthcare providers. Furthermore, the involvement of intra-organisational knowledge brokers may promote spread of innovations across health-related sectors [38]. A recent review described knowledge brokers as intermediaries to exchange knowledge across the varying stakeholders and settings, but demonstrated that the availability of evidence about their effectiveness within healthcare is limited so far [39]. In line with other studies, we found a gap in discharge [40, 41]. Unnecessary prolonged hospitalisation might be partially reduced by applying structured discharge planning, or by using an integrated discharge team [42].

The multicentre design strengthens conclusions and provides more generalisability of results. To our knowledge, the national comparison of interdepartmental spread between hospitals that had previously followed a QI programme, and those that had not, is unique. A limitation might be that the data abstractor was not blinded. We do not have any sign, however, that this has led to data manipulation, given the independent position. Otherwise, the fact that the data were extracted by one and the same person can be looked upon as a strength, as it guaranteed a standardised approach for the audit. Retrospective review of medical records has methodological limitations. The adoption of ERAS could have been underestimated, because not all elements could be collected retrospectively. It is possible that we had inadequate power to detect significant differences. However, given the 95% CIs, we would have been able to detect a clinically relevant effect of more than 1 day favouring the intervention group [43].

In conclusion, we found neither statistically significant nor clinically relevant differences in the time to FR, TLOS, or the degree of local implementation of ERAS, between the gynaecology departments of hospitals that previously followed a QI project for colorectal surgery and the hospitals that did not. Interdepartmental spread of universal evidence-based innovations within organisations seems to be restricted. Further research is required to provide potential solutions to promote knowledge sharing within hospital walls and to extend the initial positive effects of QI projects to different settings.
